# Impact of programmed cell death protein 1 inhibitor therapy on the survival of patients with advanced or recurrent uterine cancers: a meta-analysis

**DOI:** 10.3389/fimmu.2024.1331994

**Published:** 2024-03-18

**Authors:** Keng-Wei Liang, Liang-Jou Chen, Chun-Hao Wang, Kevin Sheng-Kai Ma, Li-Hsin Hsia, Po-Hui Wang

**Affiliations:** ^1^ Institute of Medicine and School of Medicine, Chung Shan Medical University, Taichung, Taiwan; ^2^ Department of Medical Imaging, Chung Shan Medical University Hospital, Taichung, Taiwan; ^3^ School of Medicine, Kaohsiung Medical University, Kaohsiung, Taiwan; ^4^ Department of Medicine, National Taiwan University, Taipei, Taiwan; ^5^ Department of Epidemiology, Harvard T.H. Chan School of Public Health, Boston, MA, United States; ^6^ Center for Global Health, Perelman School of Medicine, University of Pennsylvania, Philadelphia, PA, United States; ^7^ Division of Pharmacoepidemiology and Pharmacoeconomics, Department of Medicine, Brigham and Women's Hospital, Harvard Medical School, Boston, MA, United States; ^8^ Department of Obstetrics and Gynecology, Chung Shan Medical University Hospital, Taichung, Taiwan

**Keywords:** uterine cancer, PD-1 inhibitors, survival, adverse events, meta-analysis

## Abstract

**Introduction:**

No prior meta-analysis has investigated the impact of programmed cell death protein 1 (PD-1) inhibitor therapy on survival outcomes in patients with advanced or recurrent uterine cancers (including both corpus and cervical cancers).

**Methods:**

A comprehensive search of PubMed and Embase databases was conducted, covering the past 10 years (up to August 2023) and encompassing all clinical research related to uterine cancer. Five randomized controlled trials and one cohort study met the inclusion criteria and were included in the meta-analysis. Data on patient demographics, clinical characteristics, treatment regimens, and survival outcomes were extracted. Hazard ratios (HRs) for overall survival (OS) and progression-free survival (PFS), as well as the relative risk of grade 3 or higher adverse events, were pooled using random-effects models.

**Results:**

Patients receiving PD-1 inhibitors had better OS (HR, 0.65, 95% CI, 0.59–0.72; P<.001) and PFS (HR, 0.59, 95% CI, 0.49–0.70; P<.001) than those receiving variable non-PD-1 inhibitor therapies among 3452 uterine cancer patients. The leave-one-out meta-analysis of the HR of OS showed no individual study impact on the estimation of the overall effect size. Subgroup analysis revealed better OS in the PD-1 inhibitors use than the controls in cervical cancer (HR, 0.68, 95% CI, 0.59–0.79), endometrial cancer (HR, 0.62, 95% CI, 0.54-0.72), and pembrolizumab use (HR, 0.66, 95% CI, 0.57–0.75) subgroups. Patients with advanced cervical cancer, who had CPS > 1, receiving PD-1 inhibitors have statistically significant benefits in OS compared to controls (HR, 0.65, 95% CI, 0.53-0.80). The pooled HR for overall survival was 0.71 (95% CI, 0.60-0.82; P<.001) in patients who received PD-1 inhibitors as compared to those who did not receive PD-1 inhibitors in proficient mismatch repair (MMR) endometrial cancer patients. However, in deficient MMR patients, the HR was 0.30 (95% CI, 0.13-0.70). The relative risk of grade 3 or higher adverse events was not higher in the PD-1 inhibitor group (relative risk, 1.12, 95% CI, 0.98–1.27).

**Conclusion:**

Survival was significantly better using PD-1 inhibitor therapy than variable non-PD-1 inhibitor chemotherapies among patients with advanced or recurrent uterine cancers.

## Introduction

Biomarkers are increasingly guiding treatment decisions in immuno-oncology, including programmed cell death protein 1 (PD-1) and its ligand programmed cell death-ligand 1(PD-L1) expression, microsatellite instability (MSI) status, and mismatch repair (MMR) and total mutation burden (TMB) assessment (1–3). Specifically, MSI is a form of genetic hypermutability causing short insertion/deletion mutations in DNA, primarily at microsatellite sequences. It arises in tumor tissues due to defective DNA MMR, often caused by genetic or epigenetic inactivation of MMR pathway proteins (MLH1, MSH2, MSH6, PMS2) (4–6). The KEYNOTE-158 pembrolizumab trial established a ≥10 mutations per million bases cutoff for high TMB (7). Notably, analysis of 16,300 gynecologic cancer samples revealed significantly lower expression of PD-1/PD-L1, MSI-high (MSI-H), or high TMB in ovarian cancers compared to uterine cervical and corpus cancers (8). Strikingly, uterine corpus cancer harbors a much higher prevalence of MSI-H/dMMR (17.7%) compared to ovarian cancer (only 1.1%). Similarly, PD-1 expression is significantly higher in uterine cervical cancer (38.3%) than in ovarian cancer (7.8%). Additionally, high TMB is observed in both cervical (21.1%) and uterine corpus cancer (19.7%), further contrasting with the low rate in ovarian cancer. These stark disparities in potential response biomarkers suggest that immune checkpoint inhibitors (ICIs) might hold greater promise for treating uterine cervical and corpus cancers compared to ovarian cancer.

Uterine cancers comprise two distinct types: cervical cancer and corpus cancer. The vast majority of corpus cancers, originating from the endometrium, are adenocarcinomas, commonly called endometrial cancer (9). GLOBOCAN 2020 paints a concerning picture: cervical cancer ranks as the fourth most common cancer among women globally and second in developing countries, claiming the top spot for gynecological cancer fatalities. Uterine corpus cancer follows closely behind, ranking sixth most common and second most diagnosed gynecological malignancy worldwide. Tragically, nearly 440,000 women succumbed to these cancers in 2020 alone (10).

Although conventional therapies like surgery, chemotherapy, and radiotherapy have progressed, survival for advanced or recurrent uterine cancers remains grim. For metastatic or recurrent cervical cancer, options beyond first-line chemotherapy and bevacizumab are scarce. Second-line chemotherapy provides limited benefit, with modest response rates ranging from 15-20% (11, 12). Conversely, about 67% of endometrial cancer cases are low-grade and early-stage, boasting an impressive 81% five-year survival rate (13). However, the outlook worsens for advanced stages, with most patients progressing within a year. Alarmingly, both incidence and mortality from endometrial cancer are rising, and this trend shows no signs of slowing (14). Targeted therapies offer a ray of hope for these challenging “hot tumors” characterized by high TMB. These revolutionary approaches have transformed the treatment landscape for advanced or recurrent cervical and endometrial cancers, potentially improving outcomes for patients facing limited options. Immunotherapy, particularly with PD-1 inhibitors, has emerged as a promising weapon against cervical cancer and other malignancies (15). Studies show that 26% of patients with advanced, recurrent, PD-L1-positive endometrial carcinoma achieve remission or stabilization using these drugs (16). Additionally, a non-randomized study suggests potential antitumor activity of pembrolizumab, a specific PD-1 inhibitor, across various tumors (7). While pembrolizumab received United States Food and Drug Administration approval for advanced cervical cancer in June 2018, no meta-analysis has evaluated the impact of PD-1 inhibitors on survival outcomes across the entire spectrum of advanced or recurrent uterine cancers, encompassing both endometrial and cervical types. This meta-analysis aims to fill this gap by investigating whether PD-1 inhibitor therapy significantly improves patient survival in this population.

## Materials and methods

### Search strategy and eligibility criteria

This study was exempt from institutional review board approval and adhered to the Preferred Reporting Items for Systematic Reviews and Meta-Analyses (PRISMA) guidelines (17). A systematic review was conducted by searching the PubMed and Embase databases for clinical trials involving women with uterine cancer published in the past ten years, up to August 2023. The following search terms were used: uterine cancer, cancer of uterus, cervical cancer, uterine cervical cancer, cancer of uterine cervix, endometrial cancer, programmed cell death 1, pembrolizumab, keytruda. The detailed search strategy for PubMed is provided in the supplementary file. After screening titles and abstracts of English-language literature, two authors (P.H.W. and K.W.L.) independently reviewed full-text articles, extracted data, and assessed quality. Disagreements were resolved through consensus meetings. The inclusion criteria for full-text review were: (1) enrollment of women with metastatic, advanced, or recurrent uterine corpus or cervical cancers who received PD-1 inhibitor treatment; (2) comparison of overall survival (OS) or progression-free survival (PFS) between patients receiving PD-1 inhibitors and those receiving no PD-1 inhibitors; (3) provision of a survival analysis model for hazard ratio (HR) comparison accounting for censoring and unequal follow-up between the two groups; and (4) follow-up of at least 24 months based on survival plotting in both groups. Studies were excluded if they were: (1) clinical trials without formal published articles; (2) articles that were not randomized controlled trials or non-cohort studies focused on survival analysis; or (3) clinical studies that only reported response rates but not survival analysis.

### Quality assessment, data extraction and outcomes of interest

The quality of the included studies was assessed using the appropriate tools from the US National Institutes of Health Quality Assessment of controlled intervention studies, observational cohort studies, and cross-sectional studies (18). Two reviewers independently extracted data from eligible studies and entered it into a standardized form. This data included the first author, publication year, trial acronym, study design, cancer types, number of patients receiving PD-1 inhibitors versus those without them, treatment and control group regimens, participant age, median follow-up duration, median progression-free survival (PFS) and overall survival (OS) in months, and hazard ratios (HRs) with 95% confidence intervals (CIs) for OS and PFS. Additionally, grade 3-5 adverse events were compared between PD-1 inhibitor and non-PD-1 inhibitor treatment groups, as reported using the National Cancer Institute Common Terminology Criteria for Adverse Events, version 4.0 (19).

### Endpoints

The primary outcomes of interest were the HR comparisons of OS and PFS between patients receiving PD-1 inhibitor treatment and those receiving no PD-1 inhibitor treatment. The secondary endpoints were the incidences of treatment-related adverse events.

### Statistical analyses

All data syntheses and analyses were conducted using Stata/MP version 17.0 software (StataCorp, College Station, TX, USA). Recognizing potential variability in study effects, a random-effects model was employed to separately estimate the pooled hazard ratio (HR) with 95% confidence intervals (CI) for patient survival and the pooled incidence rate and relative risk with 95% CI for adverse events of grade 3 or higher. Statistical significance was assessed with a p-value of 0.05 or less. For both outcomes, required data not reported in the original studies were transformed following established methods (20). Subgroup analyses were conducted based on cancer type, treatment regimen, permbrolizumab use, combined positive score (CPS), and MMR status. Data homogeneity was assessed using Cochran’s chi-square Q test and I² statistic for each outcome. Sensitivity analysis via leave-one-out meta-analysis examined the stability of results for both outcomes. Potential publication bias for each outcome was evaluated through funnel plots and Egger’s test, with p < 0.1 indicating significance.

## Results

### Studies conforming to the inclusion criteria

**Figure 1:**
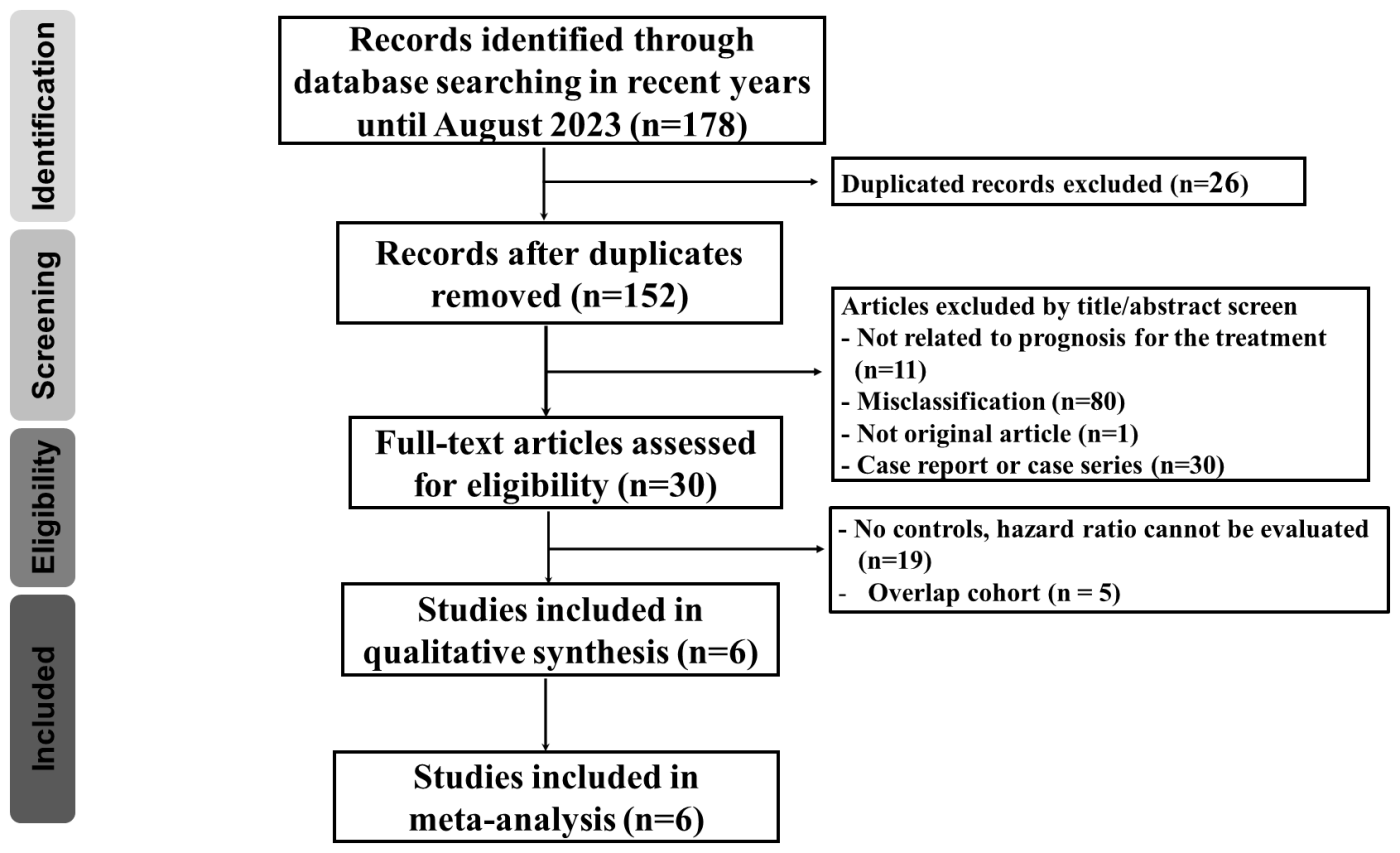
Flow diagram demonstrating the process of publication review and the inclusion of eligible studies.

Our search strategy identified 178 articles associated with uterine cancers. After removing duplicates (n = 26), 152 articles remained. Title and abstract screening excluded 122 articles, leaving 30 for full-text review. Among these, 24 were excluded due to no controls (n = 19) and overlap with previously included cohorts (n = 5). The detailed enrollment flowchart is shown in **Figure 1**. Six articles were ultimately included for final analysis (21–26). **Table 1** summarizes their basic characteristics. A total of 3,452 patients were enrolled. Five of the six studies were randomized controlled trials. Two of these focused on patients with cervical cancer, while the remaining three primarily focused on endometrial cancer. The remaining study was a cohort study that investigated endometrial cancer patient survival. The HR for OS and for PFS can be assessed in five of the six studies, respectively. One study reported survival outcomes stratified by mismatch repair (MMR) status only; therefore, pooled HRs were calculated separately for each group (26). No publications were excluded due to quality concerns, as assessed using the National Institutes of Health quality assessment tool. Four studies were rated “good” and two were rated “fair” in overall quality. The detailed quality assessment is presented in **Table 2**. Among the PD-1 inhibitor treatment regimens, three studies used pembrolizumab, while cemiplimab, nivolumab, and dostarlimab were used in one study each. Adjuvant monotherapy with cemiplimab was used in one study, while combined therapy was prescribed in the remaining five PD-1 inhibitor studies. Chemotherapy was used in all control groups for comparison. Adverse events were reported in five randomized controlled trials. The patient population included 1,225 cervical cancer patients and 2,227 endometrial cancer patients. Subgroup analyses for survival were performed based on cancer type (cervical vs. endometrial). In cervical cancer, pooled HRs were assessed based on CPS, while in endometrial cancer, they were assessed based on MMR status.

**Table 1:** Characteristics of the enrolled studies.

Study, years	Design	Cancer type	Number (T/C)^a^	Regimen (T)	Regimen (C)	Age (year)	FU duration (mo)^b^	Median PFS (mo)	Median OS (mo)
**Colombo et al.** (21)	RCT	Cervix	308/309	Pembro + CT ± BEV	Placebo + CT ± BEV	51 vs 50	22.0	10.4 vs 8.2	24.4 vs 16.3-16.5
**Tewari et al.** (22)	RCT	Cervix	304/304	Cemiplimab	Single-agent CT^c^	51 vs 50	18.2	2.8 vs 2.9	12.0 vs 8.5
**Liao et al.** (23)	Cohort study	EM	52/41	NIVO + BEV	PTX + BEV	46.7 vs 45.2	NA	NA	33.2 vs 21.8
**Makker et al.** (24)	RCT	EM	411/416	Pembro + Lenva	DOX or PTX	64 vs 65	12.2 vs 10.7	7.2 vs 3.8	18.7 vs 11.9
**Mirza et al.** (25)	RCT	EM	245/249	Dostarlimab + CT	Placebo + CT	64 vs 65	25.4	NA	NA
**Eskander et al.** (26)	RCT	EM	(d)^*^ 112/113	Pembro + CT	Placebo + CT	67 vs 66	12	NR vs 7.6	NA
			(p)**^†^** 293/295	Pembro + CT	Placebo + CT	66 vs 65	7.9	13.1 vs 8.7	NA

a Case numbers in programmed cell death protein 1 inhibitors group and control group, respectively

b Represented mean or median as reported

c Variable regimen includes pemetrexed, gemcitabine, topotecan, irinotean, vinorelbine … etc

^*^(d) Data stands for patient number in the deficient mismatch repair group.

^†^(p) Data stands for patient number in the proficient mismatch repair group.

T, treatment group; C, control group; FU, follow up; mo, months, PFS, progression-free survival; OS, overall survival; RCT, randomized control trial; EM, endometrium; Pembro, Pembrolizumab; Lenva, Lenvatinib; CT, chemotherapy; BEV, Bevacizumab; NIVO, nivolumab; DOX, Doxorubicin; PTX, Paclitaxel; NA, not available; NR, not reached.

**Table 2:** Quality Assessment of the Included Studies.

Study	NIH quality assessment tool for controlled intervention studies														
	Q1	Q2	Q3	Q4	Q5	Q6	Q7	Q8	Q9	Q10	Q11	Q12	Q13	Q14	Quality rating
**Colombo et al.** (21)	✓	✓	✓	✓	✓	✓	✓	✓	✕	✓	✓	✓	✓	✓	**Good**
**Tewari et al.** (22)	✓	✓	✓	✕	✕	✓	✓	✓	✕	✓	✓	✓	✓	✓	**Good**
**Makker et al.** (24)	✓	✓	✓	✕	✕	✓	NR	NR	CD	✓	✓	✓	✓	✓	**Fair**
**Mirza et al.** (25)	✓	✓	✓	✓	✓	✓	✓	✓	✓	✓	✓	✓	✓	✓	**Good**
**Eskander et al.** (26)	✓	✓	✓	✓	✓	✓	✓	✓	✓	✓	✓	✓	✓	✓	**Good**
	NIH quality assessment tool for observational cohort and cross-sectional studies														
	Q1	Q2	Q3	Q4	Q5	Q6	Q7	Q8	Q9	Q10	Q11	Q12	Q13	Q14	Quality rating
**Liao et al.** (23)	✓	✓	✓	✓	✕	✓	✓	NA	✓	✓	✓	✕	NR	✕	**Fair**

NIH, National Institutes of Health; ✓ = Criterion was met; x = Criterion was not met; NR, not reported; CD, cannot determine; NA, not applicable. Good = met 7–9 criteria; fair = met 4–6 criteria; poor = met 0–3 criteria.

Questions for controlled intervention studies:

Q1 = Was the study described as randomized, a randomized trial, a randomized clinical trial, or an RCT?; Q2 = Was the method of randomization adequate (i.e., use of randomly generated assignment)? Q3 = Was the treatment allocation concealed (so that assignments could not be predicted)?; Q4 = Were study participants and providers blinded to treatment group assignment?; Q5 = Were the people assessing the outcomes blinded to the participants’ group assignments?; Q6 = Were the groups similar at baseline on important characteristics that could affect outcomes (e.g., demographics, risk factors, co-morbid conditions)?; Q7 = Was the overall drop-out rate from the study at endpoint 20% or lower of the number allocated to treatment?; Q8 = Was the differential drop-out rate (between treatment groups) at endpoint 15 percentage points or lower?; Q9 = Was there high adherence to the intervention protocols for each treatment group?; Q10 = Were other interventions avoided or similar in the groups (e.g., similar background treatments)?; Q11 = Were outcomes assessed using valid and reliable measures, implemented consistently across all study participants?; Q12 = Did the authors report that the sample size was sufficiently large to be able to detect a difference in the main outcome between groups with at least 80% power?; Q13 = Were outcomes reported or subgroups analyzed prespecified (i.e., identified before analyses were conducted)?; Q14 = Were all randomized participants analyzed in the group to which they were originally assigned, i.e., did they use an intention-to-treat analysis?

For observational cohort and cross-sectional studies

Q1 = Was the research question or objective in this paper clearly stated?; Q2 = Was the study population clearly Was the participation rate of eligible persons at least 50%?; Q4 = Were all the subjects selected or recruited from the same or similar populations (including the same time period)? Were inclusion and exclusion criteria for being in the study prespecified and applied uniformly to all participants?; Q5 = Was a sample size justification, power description, or variance and effect estimates provided?; Q6 = For the analyses in this paper, were the exposure(s) of interest measured prior to the outcome(s) being measured?; Q7 = Was the timeframe sufficient so that one could reasonably expect to see an association between exposure and outcome if it existed?; Q8 = For exposures that can vary in amount or level, did the study examine different levels of the exposure as related to the outcome (e.g., categories of exposure, or exposure measured as continuous variable)?; Q9 = Were the exposure measures (independent variables) clearly defined, valid, reliable, and implemented consistently across all study participants?; Q10 = Was the exposure(s) assessed more than once over time?; Q11 = Were the outcome measures (dependent variables) clearly defined, valid, reliable, and implemented consistently across all study participants?; Q12 = Were the outcome assessors blinded to the exposure status of participants?; Q13 = Was loss to follow-up after baseline 20% or less?; Q14 = Were key potential confounding variables measured and adjusted statistically for their impact on the relationship between exposure(s) and outcome(s)?

### Impact of PD-1 inhibitor therapy on survival

**Figure 2:**
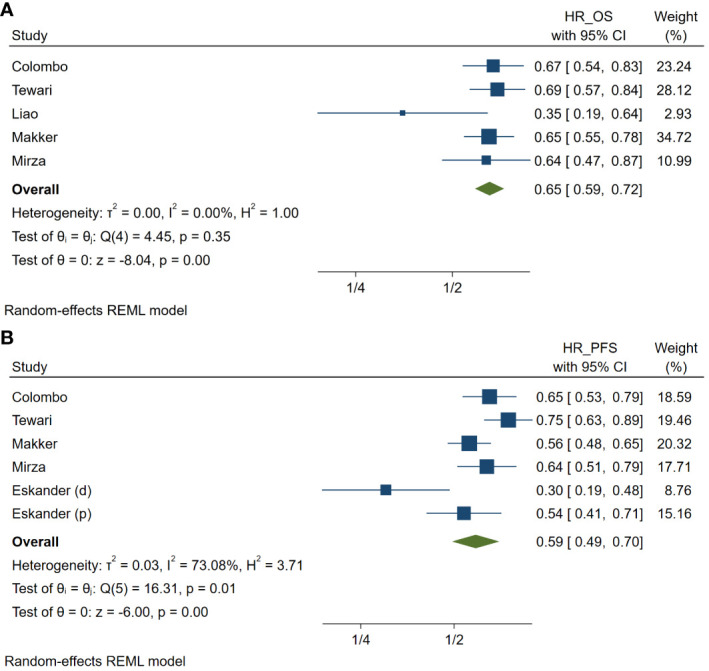
Meta-analysis of studies on the prognosis in patients treated with programmed cell death protein 1 (PD-1) inhibitors compared with those treated with variable non-PD-1 inhibitor therapies in hazard ratio (HR) of overall survival **(A)** and of progression-free survival **(B)** in patients with uterine cancers. HR_OS, hazard ratio of overall survival; HR_PFS, hazard ratio of progression free survival. (d), cohort with deficient mismatch repair; (p), cohort with proficient mismatch repair.

Meta-analysis revealed that patients with uterine cancer who received PD-1 inhibitors had a better OS compared to patients in the diverse non-PD-1 inhibitor chemotherapy group across five studies. The pooled HR for OS was 0.65 (95% CI: 0.59–0.72, p <.001; I² = 10.02%, Cochran’s p = 0.35) (**Figure 2A**). Similarly, the pooled HR for PFS across five studies was 0.59 (95% CI: 0.49–0.70, p <.001; I² = 73.08%, Cochran’s p = 0.01) (**Figure 2B**).

### Publication bias and sensitivity analysis

**Figure 3:**
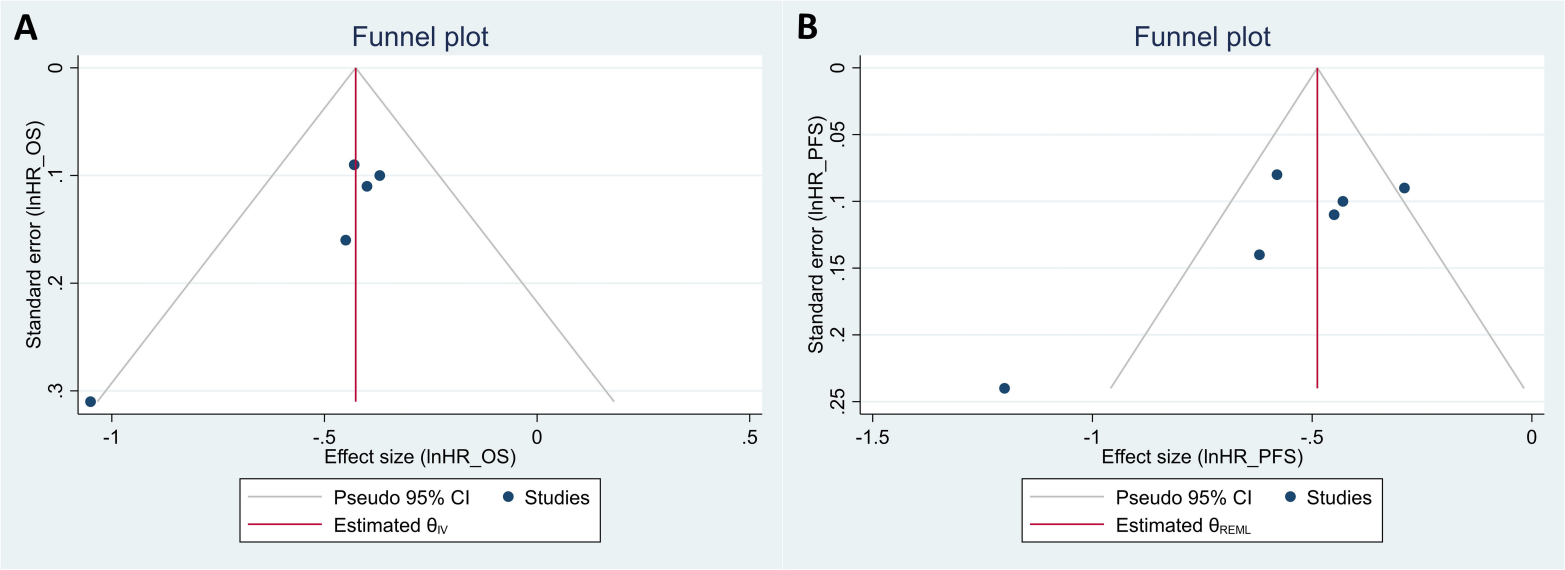
Funnel plots for the evaluation of publication bias in studies regarding the hazard ratio (HR) of overall survival **(A)** and of progression-free survival **(B)** in patients with uterine cancers who were treated with programmed cell death protein 1 (PD-1) inhibitors compared with those treated with variable non-PD-1 inhibitor therapies.

The Egger’s test indicated potential publication bias for both OS and PFS (P = 0.066 and P = 0.009, respectively). Funnel plots for both outcomes also show some asymmetry, further suggesting possible publication bias (**Figures 3A, B**). However, leave-one-out sensitivity analyses of the HR for OS and PFS revealed that no single study significantly impacted the overall effect size estimates (**Supplementary Figure 1A, B**).

### Subgroup analysis

The pooled HR for OS in patients with cervical cancer who received PD-1 inhibitors was 0.68 (95% CI: 0.59–0.79, p <.001; I² = 0%, Cochran’s p =.84; **Supplementary Figure 2A**) (21, 22). Similarly, the pooled HR for OS in patients with endometrial cancer who received PD-1 inhibitors was 0.62 (95% CI: 0.54-0.72, p <.001; I² = 0%, Cochran’s p =.16) (**Supplementary Figure 2A**) (23–25). Among patients with uterine cancers, pembrolizumab, a common PD-1 inhibitor, showed a pooled HR of 0.66 for OS (95% CI: 0.57–0.75, p <.001; **Supplementary Figure 2B**) (21, 24). Its pooled HR for PFS was 0.53 (95% CI: 0.41-0.67, p <.001; **Supplementary Figure 2C**).

Focusing on cervical cancer, patients with a CPS greater than 1 who received PD-1 inhibitor therapy had better OS compared to those who did not (pooled HR: 0.65, 95% CI: 0.53-0.80; p <.001; **Supplementary Figure 2D**) (21, 22). However, for patients with CPS less than 1, the pooled HR for OS showed no significant improvement with PD-1 inhibitors compared to non-PD-1 inhibitor therapies (pooled HR = 0.99, 95% CI = 0.67–1.47; p = 0.95; **Supplementary Figure 2E**).

In endometrial cancer, considering mismatch repair MMR status, the pooled HR for OS was 0.71 (95% CI: 0.60-0.82; p <.001; **Supplementary Figure 2F**) in pMMR patients who received PD-1 inhibitors compared to those who did not (24, 25). For dMMR patients, the HR for OS was 0.30 (95% CI: 0.13-0.70) in the PD-1 inhibitor group (25). Overall, the pooled HR for OS in all endometrial cancer patients receiving PD-1 inhibitors was 0.69 (95% CI: 0.59–0.80; p = 0.05; **Supplementary Figure 2F**). Notably, the reduction in HR for OS appeared more pronounced in dMMR patients (**Supplementary Figure 2F**). Similarly, the pooled HR for PFS was 0.63 (95% CI: 0.52-0.75; p <.001; **Supplementary Figure 2G**) in pMMR patients treated with PD-1 inhibitors compared to controls (24–26). In dMMR patients, the pooled HR for PFS was 0.29 (95% CI: 0.20-0.42; p <.001) with PD-1 inhibitors (25, 26). The overall pooled HR for PFS in all endometrial cancer patients receiving PD-1 inhibitors was 0.49 (95% CI: 0.34-0.70; p <.001). The HR reduction for PFS also seemed more significant in dMMR patients.

### Adverse events

Five randomized controlled studies reported adverse events. Among patients treated with PD-1 inhibitors, 1,143 out of 1,639 (69.7%, 95% CI: 67.5%–72.0%) experienced cumulative grade 3–5 adverse events. The most common grade 3-5 adverse events were hypertension (205 patients), anemia (193 patients), neutropenia (72 patients), urinary tract infection (59 patients), and weight loss (44 patients). The aggregated incidence rate of grade 3–5 adverse events in the PD-1 inhibitor treatment group was 69%, with substantial heterogeneity (95% CI: 53%–85%, p <.001; I² = 98.43%, Cochran’s p <.001) (**Supplementary Figure 3A**). The pooled relative risk of grade 3–5 adverse events for PD-1 inhibitors compared to controls was 1.12 (95% CI: 0.98–1.27), indicating no significant difference in adverse events between the PD-1 inhibitor and non-PD-1 inhibitor groups (**Supplementary Figure 3B**).

## Discussion

This meta-analysis demonstrates that PD-1 inhibitor therapy is significantly more effective than other standard treatments, such as conventional chemotherapy and targeted therapies, for patients with advanced or recurrent uterine cancers. Patients treated with PD-1 inhibitors experienced a remarkable 35% reduction in the risk of death and a 41% reduction in disease progression compared to those on other treatments. Subgroup analysis showed these positive effects across both endometrial and cervical cancers. Specifically, PD-1 inhibitor therapy improved overall survival by 38% in endometrial cancer patients, with an associated 32% reduction in mortality in patients with cervical cancer.

Research suggests that PD-1 inhibitors may offer a significant survival benefit for patients with advanced uterine cancer, particularly those with mismatch repair (dMMR) or high microsatellite instability (MSI-H). Studies by Mirza et al., Makker et al., and Eskander et al. show a clear reduction in overall survival and progression-free survival risk when dMMR/MSI-H patients receive PD-1 inhibitors compared to non-PD-1 treatments (24, 25). This contrasts with findings by Liao et al., where dMMR patients treated with chemotherapy experienced worse outcomes than their pMMR counterparts, but showed similar survival benefits with PD-1 inhibitors (23). These results suggest that while dMMR/MSI-H patients might have the poorest response to chemotherapy, they could potentially experience significant improvement with PD-1 therapy. Similarly, pembrolizumab monotherapy appears to be less effective in patients with microsatellite stable (MSS) or pMMR disease compared to those with MSI-H or dMMR disease (7). Similar trends are observed in cervical cancer, where PD-1 inhibitors demonstrate improved survival only in patients with high combined positive score (CPS), indicating greater tumor mutational burden and potential immune response activation. These findings highlight the critical role of genomic expression profiles in predicting responses to PD-1 inhibitors. While the current evidence is promising, further research with larger studies and pairwise comparisons based on genomic expression is crucial for validation. This will provide valuable guidance for healthcare systems considering expanding access to PD-1 inhibitors for specific patient subgroups (24, 25).

Four PD-1 inhibitors (pembrolizumab, cemiplimab, nivolumab, and dostarlimab) were used for treating advanced uterine cancer in the included study. These drugs work by blocking the interaction between PD-1 and its ligands, unleashing the immune system’s cytotoxic forces to attack and destroy tumors (27). Subgroup analysis revealed pembrolizumab’s potential to improve overall survival. Studies suggest its use with chemotherapy as a first-line treatment for recurrent or metastatic head and neck squamous cell carcinoma (HNSCC) and monotherapy for PD-L1-positive HNSCC (28). Additionally, Makker et al. found that lenvatinib combined with pembrolizumab significantly improved overall survival in advanced endometrial cancer compared to chemotherapy alone (29). However, lenvatinib alone has limited efficacy in recurrent cases (30). Crucially, the included studies employed diverse treatment regimens, highlighting the need for further research to determine the optimal approach for each patient subgroup. This multifaceted approach makes pinpointing the most effective combination challenging. Further investigation is also needed in several areas. While initial and mid-term results are encouraging, long-term monitoring of both safety and efficacy is crucial for optimal patient care and long-term policy decisions regarding PD-1 inhibitor use. Additionally, the high cost of these drugs raises concerns about affordability and equitable access within healthcare systems. Cost-effectiveness analyses are essential to explore strategies for ensuring broader access to these potentially life-extending therapies.

Almost 70% of uterine cancer patients receiving PD-1 inhibitors faced serious side effects (grade 3-5), but the exact percentage varied across studies. These side effects, including high blood pressure and anemia, were generally similar to those experienced by patients on other treatments. Importantly, the risk of serious side effects remained comparable between the PD-1 and non-PD-1 groups (pooled risk rate estimate: 1.12, 95% CI: 0.98–1.27). However, one study suggests that cemiplimab monotherapy, a specific PD-1 inhibitor, might offer lower rates of these side effects compared to chemotherapy (22).

There were some limitations in this study. First, different regimens were prescribed in the treatment and control groups among these studies. The heterogeneity exists for the assessment of PFS in uterine cancers and the assessment of PFS in pembrolizumab treatment. However, we also did a sensitivity analysis with leave-one-out meta-analysis to test the robustness of our findings and showed that no individual study had a statistically significant impact on the estimation of the overall effect size. Second, only six researches were candidates for patient survival in the meta-analysis with a small sample size after we tried our best to search the extensive literature. Third, the results of the Egger test and the asymmetry of the funnel plot for overall survival and progression-free survival suggest that some publication bias may exist. Therefore, the analysis results should be interpreted with caution. There may be some heterogeneities in the included studies, such as cancer type, treatment regimen, study design, and genome mutation status. However, a subgroup analysis considering these covariates is not yet possible based on the current database search results. Future analysis is warranted after more treatment results are reported. Last, unpublished articles were excluded, only English-language studies were included, and studies with negative results were less likely to be published. Our meta-analysis only included published researches may present a publication bias.

## Conclusions

This meta-analysis suggests that PD-1 inhibitor therapy holds promise for significantly improving survival outcomes in patients with advanced uterine cancers, while offering similar rates of severe side effects compared to traditional treatments like chemotherapy. Importantly, the analysis observed benefits in both cervical and endometrial cancer patients, including increased overall and progression-free survival. Notably, the analysis also revealed a reduced risk of death for cervical cancer patients with high CPS scores and more pronounced survival improvements for endometrial cancer patients with mismatch repair deficiency.

However, it is crucial to acknowledge that while this study provides novel and promising insights, the sample size remains relatively small. As more robust randomized and cohort studies emerge, further research is needed to solidify these findings and provide stronger support for the broader application of PD-1 inhibitors in uterine cancer treatment.

## Data availability statement

The raw data supporting the conclusions of this article will be made available by the authors, without undue reservation.

## Ethics statement

Ethical approval was not required for the study involving humans in accordance with the local legislation and institutional requirements. Written informed consent to participate in this study was not required from the participants or the participants’ legal guardians/next of kin in accordance with the national legislation and the institutional requirements.

## Author contributions

K-WL: Formal analysis, Writing – original draft, Methodology, Investigation, Data curation. L-JC: Writing – original draft, Investigation, Formal analysis. C-HW: Writing – original draft, Investigation, Data curation. KM: Writing – review & editing. L-HH: Writing – review & editing, Conceptualization. P-HW: Writing – review & editing, Methodology, Formal Analysis, Conceptualization.
