# Supplementary material for: Impact of programmed cell death protein 1 inhibitor therapy on the survival of patients with advanced or recurrent uterine cancers: a meta-analysis
Source: Front Immunol. 2024 Mar 18;15:1331994. doi: 10.3389/fimmu.2024.1331994 (PMC10982406; doi:10.3389/fimmu.2024.1331994)

**Supplementary Material**

**Detailed Search Process**

The search process is represented by the search strategy in PubMed.

((((((Uterine cancer[Title/Abstract]) OR (Cancer, uterus[Title/Abstract])) OR (Cervical cancer[Title/Abstract])) OR (Uterine cervical cancer[Title/Abstract])) OR (Cancer, uterine cervix[Title/Abstract])) OR (Endometrial cancer[Title/Abstract]) AND ((y_10[Filter]) AND (fha[Filter]) AND (clinicaltrial[Filter]) AND (female[Filter]))) AND (((Programmed cell death 1[Title/Abstract]) OR (Pembrolizumab[Title/Abstract])) OR (Keytruda[Title/Abstract]) AND ((y_10[Filter]) AND (fha[Filter]) AND (clinicaltrial[Filter]) AND (female[Filter]))) Filters: Abstract, Female, Clinical Trial, in the last 10 years.

**2.1 Supplementary Figures**

**Supplementary Figure 1** The leave-one-out meta-analysis of the hazard ratio of overall survival in advanced uterine cancer patients treated with programmed cell death protein 1(PD-1) inhibitors compared with those treated with variable non-PD-1 inhibitor therapies. HR_OS = hazard ratio of overall survival; HR_PFS, hazard ratio of progression free survival; (d), cohort with deficient mismatch repair; (p), cohort with proficient mismatch repair.


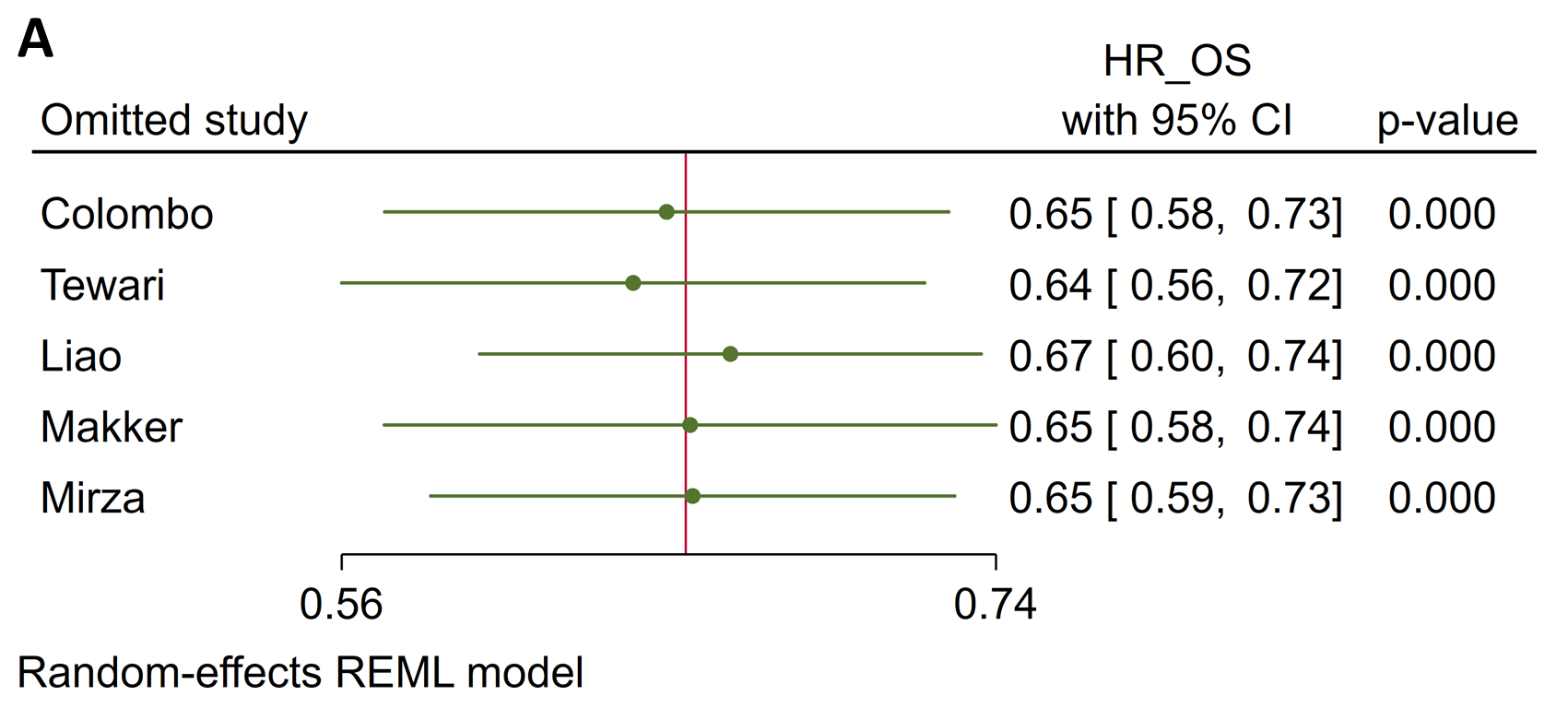

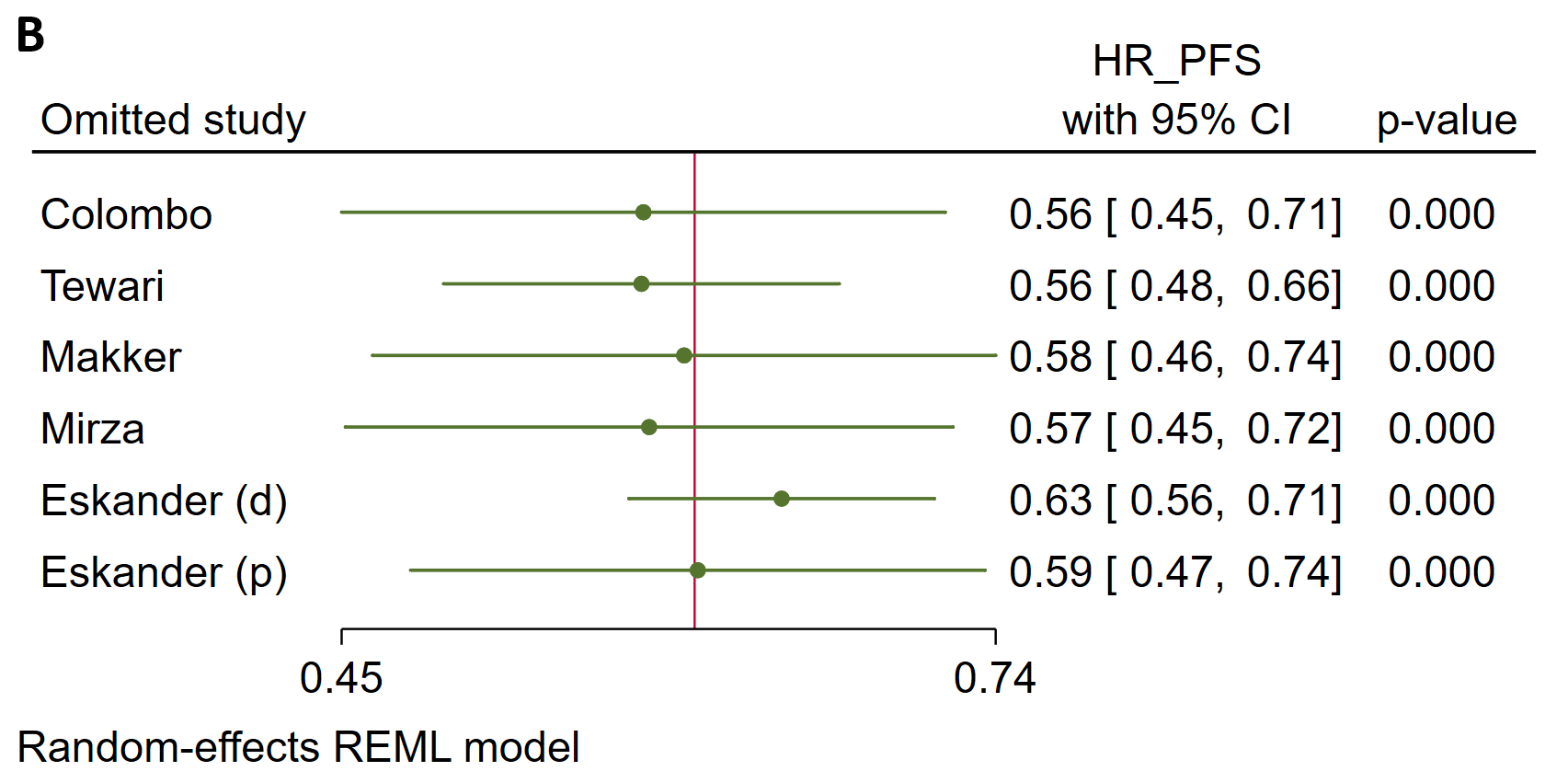


**Supplementary Figure 2** Subgroup meta-analysis of studies on the prognosis in patients treated with programmed cell death protein 1(PD-1) inhibitors compared with those treated with variable non-PD-1 inhibitor therapies in hazard ratio (HR) of overall survival in patients with cervical cancers (A), HR of overall survival in patients with endometrial cancers (B), HR of overall survival in patients treated with pembrolizumab (C), HR of progression-free survival in patients treated with pembrolizumab (D), HR of overall survival in cervical cancer patients with combined positive score ≥1 (E), and HR of overall survival in cervical cancer patients with combined positive score <1 (F). HR_OS = hazard ratio of overall survival. HR_PFS = hazard ratio of progression-free survival; (d), cohort with deficient mismatch repair; (p), cohort with proficient mismatch repair.


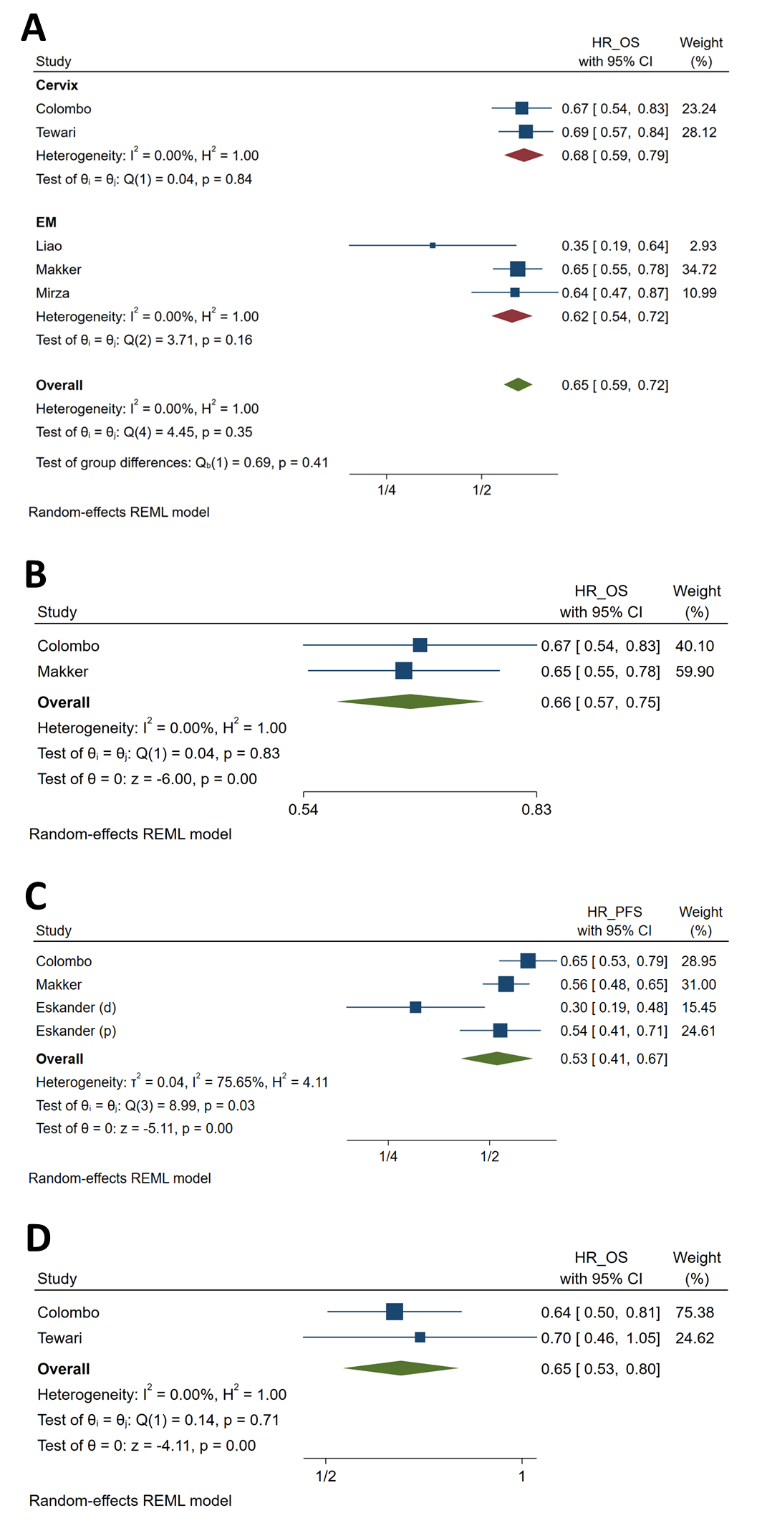

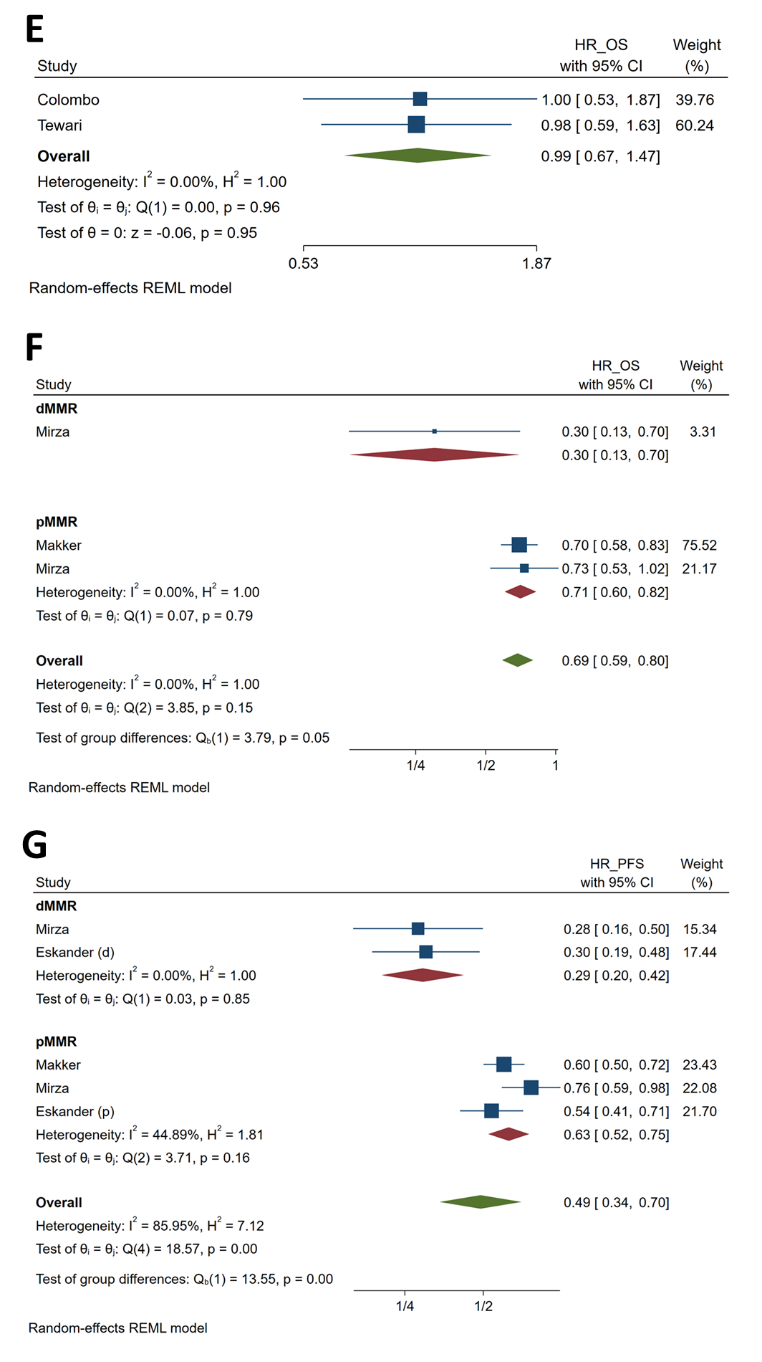


**Supplementary Figure 3** Results of the aggregated incidence rate of grade 3–5 adverse events among the programmed cell death protein 1 (PD-1) inhibitors therapy group (A) and the relative risk of grade 3–5 adverse events for PD-1 inhibitors therapy group as compared with the non-PD 1 inhibitors therapies group (B).


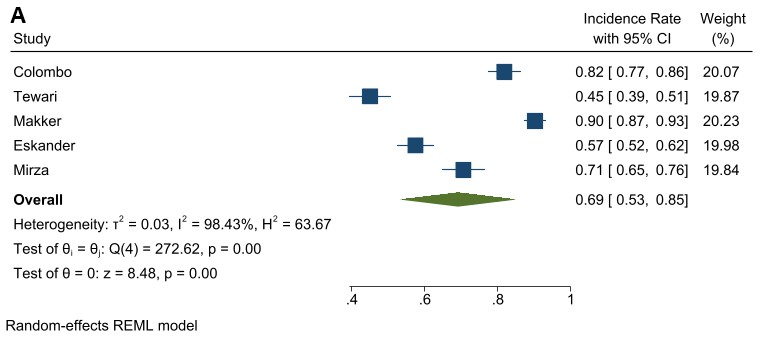


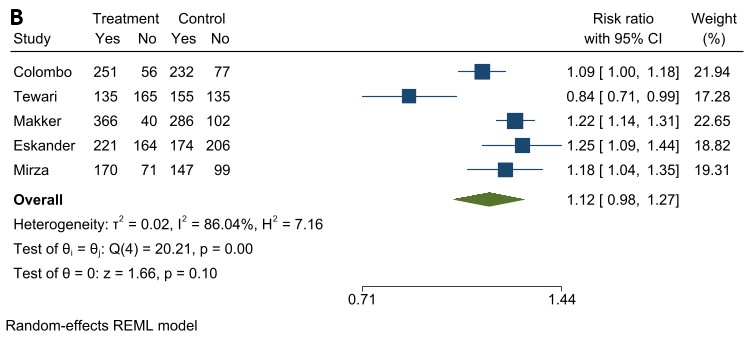

Supplement: Supplementary file 1 [file DataSheet_1.docx]
